# Anti-apoptotic Mutations Desensitize Human Pluripotent Stem Cells to Mitotic Stress and Enable Aneuploid Cell Survival

**DOI:** 10.1016/j.stemcr.2019.01.013

**Published:** 2019-02-14

**Authors:** Jing Zhang, Adam J. Hirst, Fuyu Duan, Hui Qiu, Rujin Huang, Ying Ji, Lufeng Bai, Fengzhi Zhang, Darren Robinson, Mark Jones, Le Li, Peizhe Wang, Peng Jiang, Peter W. Andrews, Ivana Barbaric, Jie Na

**Affiliations:** 1 Center for Stem Cell Biology and Regenerative Medicine, School of Medicine, Tsinghua University, Beijing 100084, China; 2 Centre for Stem Cell Biology, Department of Biomedical Science, The University of Sheffield, Sheffield S10 2TN, UK; 3 Light Microscopy Facility, Department of Biomedical Science, The University of Sheffield, Sheffield S10 2TN, UK; 4 School of Life Sciences, Tsinghua University, Beijing 100084, China

**Keywords:** human pluripotent stem cells, chromosome instability, apoptosis, BCL-XL, NOXA

## Abstract

Human pluripotent stem cells (hPSCs) are susceptible to numerical and structural chromosomal alterations during long-term culture. We show that mitotic errors occur frequently in hPSCs and that prometaphase arrest leads to very rapid apoptosis in undifferentiated but not in differentiated cells. hPSCs express high levels of proapoptotic protein NOXA in undifferentiated state. Knocking out NOXA by CRISPR or upregulation of the anti-apoptosis gene BCL-XL significantly reduced mitotic cell death, allowing the survival of aneuploid cells and the formation of teratomas significantly larger than their wild-type parental hPSCs. These results indicate that the normally low threshold of apoptosis in hPSCs can safeguard their genome integrity by clearing cells undergoing abnormal division. The amplification of *BCL2L1* on chromosome 20q11.21, a frequent mutation in hPSCs, although not directly oncogenic, reduces the sensitivity of hPSCs to damage caused by erroneous mitosis and increases the risk of gaining aneuploidy.

## Highlights

• Live-imaging study revealed frequent mitotic errors in undifferentiated hPSCs
• Undifferentiated hPSCs are highly sensitive to mitotic stress-induced apoptosis
• High NOXA level primes abnormally divided hPSCs to die
• Reducing TP53 cannot rescue mitotic stress-induced hPSC death

## Graphical Abstract

**Figure:**
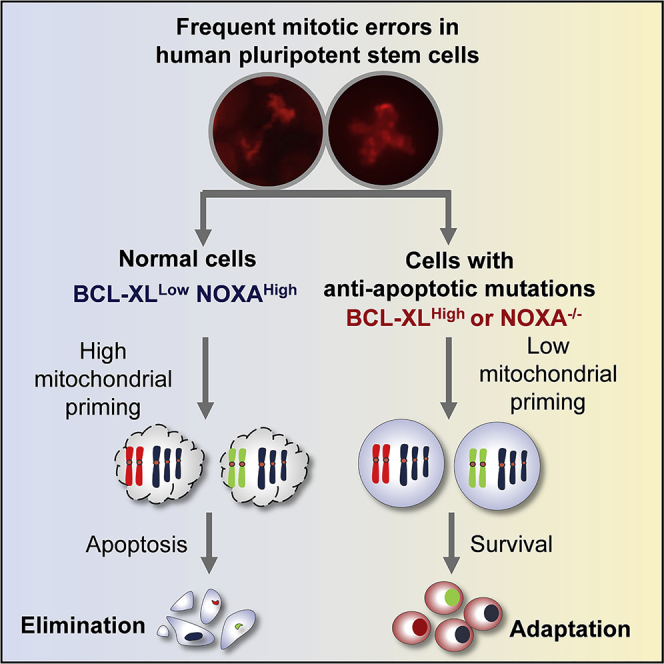

## Teaser

In this article, Na, Barbaric and colleagues showed that aberrant mitosis is common in hPSCs. A high level of NOXA, a proapoptotic protein, makes hPSCs highly sensitive to mitotic stress, thus cells with chromosomal abnormality are eliminated. Anti-apoptotic mutations, such as BCL2L1 CNV, significantly enhanced the survival ability of hPSCs and increased the chance of aneuploid cell retention.

## Introduction

For the safe use of human pluripotent stem cells (hPSCs) in regenerative medicine, they should be devoid of mutations that could render them or their differentiated progeny malignant upon transplantation into a patient (Goldring et al., 2011). One important question is whether genetic or epigenetic changes acquired during hPSC *in vitro* culture will affect the safety and efficacy of derivatives of hPSCs produced for therapeutic application (Andrews et al., 2017). While at low passage, most of the hPSC lines have normal diploid karyotype, the incidence of aneuploidy increases significantly with passage number, and gains of the whole or parts of chromosomes 1, 12, 17, and 20 are substantially more common than other changes (Amps et al., 2011, Taapken et al., 2011). Most likely, these genetic changes are selected because they confer a growth advantage (Olariu et al., 2010), which may be attributed to their ability to evade the bottlenecks that restrict the expansion of wild-type cells in culture, including mass cell death following plating, failure to re-enter the cell cycle, and the high death rate of daughter cells in incipient colonies (Barbaric et al., 2014).

The frequent appearance of hPSCs with gains of whole chromosomes suggests their susceptibility to chromosome segregation errors during mitosis. In somatic cells a key regulatory mechanism controlling accurate chromosome segregation is the mitotic checkpoint, which delays the onset of anaphase and arrests cells in prometaphase to correct the defects (Stukenberg and Burke, 2015). After prolonged prometaphase arrest, cells may either die or exit mitosis without proper chromosome separation, thereby forming tetraploid or aneuploid cells in G_1_ phase, a process termed mitotic slippage (Topham and Taylor, 2013). Cell fates following mitotic slippage include apoptosis, senescence, or re-entry into the cell cycle, with the latter often resulting in highly aberrant genomes (Topham and Taylor, 2013). The frequency of aberrant divisions in hPSCs and their behavior following the mitotic checkpoint activation is poorly characterized. High rates of death in hPSC cultures (Barbaric et al., 2014) suggest a reliance of cells on apoptosis for clearing genetically damaged cells. For example, hPSCs subjected to DNA-replication stress in S phase rapidly commit to apoptosis rather than initiate DNA repair mechanisms (Desmarais et al., 2012).

Given the important role of apoptosis in protecting the genome stability of a cell population, an increase in apoptotic threshold through overexpression of anti-apoptotic genes could provide a mechanism for survival of cells with genetic damage. This phenomenon, previously observed in cancer cells (Williams et al., 2005), may be particularly pertinent to hPSCs. In a large-scale study of karyotype and copy-number variation (CNV) in hPSCs by the International Stem Cell Initiative (ISCI), 26% of karyotypically normal hPSC lines examined contained amplifications of a small region of the long arm of chromosome 20 (20q11.21) including the *BCL2L1* gene. Subsequent studies identified increased expression levels of BCL-XL, the BCL2L1 anti-apoptotic isoform from the amplified chromosome 20q11.21 region, as an underlying cause for the enhanced survival of the CNV cells (Avery et al., 2013, Nguyen et al., 2014). However, it remains unknown how acquired overexpression of *BCL2L1* may affect the subsequent genetic stability of hPSCs.

Here we show that hPSCs commit to apoptosis rapidly in response to nocodazole-induced prometaphase arrest or following a highly aberrant cell division due to high mitochondrial priming. After differentiation, hPSCs are no longer sensitive to prometaphase arrest. The proapoptotic gene *NOXA* is responsible for the highly sensitive mitochondrial apoptosis present in hPSCs. Knockout of *NOXA* by CRISPR in hPSCs or overexpression of the anti-apoptotic protein, BCL-XL, significantly reduced cell death caused by defective mitosis. BCL-XL overexpression or the presence of the *BCL2L1* CNV had enhanced survival ability, altered mitochondrial morphology, and aneuploidy formation after perturbing mitosis. Our study reveals the vulnerability of hPSC mitosis and comprehensively assesses the biological consequence of gaining anti-apoptotic mutations in hPSC. These findings reveal an important roadmap that drives hPSC culture adaptation.

## Results

### HPSCs Are Prone to Mitotic Division Errors

**Figure 1:**
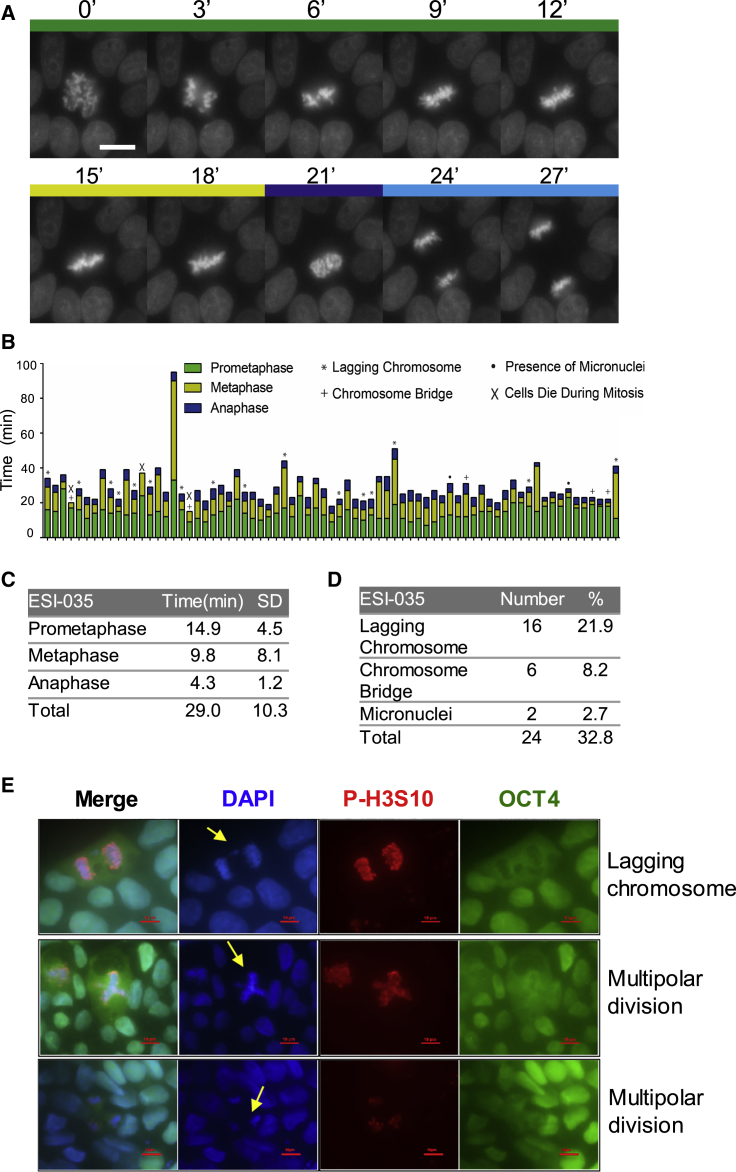
Live-Imaging Study of hPSC Mitosis (A) Selective frames from time-lapse movies of H2B-mCherry ESI-035 normal hPSC division. The time (in minutes) is indicated at the top of each frame. (B) Bar graph representation of the length of each phase of the mitosis based on live imaging of 73 individual cells pooled from three independent experiments. Prometaphase, metaphase, and anaphase are indicated by different colors, and different types of division defects are marked by indicated symbols. (C) Mean duration (minutes) of prometaphase, metaphase, and anaphase of ESI-035-H2B-mCherry cells based on time-lapse videos. (D) Frequency of defective divisions in ESI-035-H2B-mCherry cells based on time-lapse videos. (E) Abnormal mitosis in routine hPSC culture. Lagging chromosome and multipolar division are highlighted by yellow arrows (DNA in blue, phosphorylated H3S10 in red, OCT4 in green).

To analyze chromosomal dynamics during hPSC mitosis, we performed time-lapse imaging of karyotypically normal cells expressing histone H2B-mCherry (Figure 1A and Video S1). In 73 cells analyzed by time-lapse microscopy, the progression from chromosome condensation in prophase to completion of cytokinesis lasted about 29 min (Figures 1B and 1C), with prometaphase lasting 14.9 ± 4.5 min, metaphase 9.8 ± 8.1 min, and anaphase 4.3 ± 1.2 min (Figure 1C). Abnormal mitosis was observed in over 30% of cells, and these included lagging chromosomes (22%) and chromosomal bridges (8%) (Figures 1B–1E and Video S2). Cell death during division could also be seen (Figure 1B). We also examined fixed hPSCs and found that cells in mitosis displayed similar errors as well as multipolar division (Figure 1E).

Video S1. Time-Lapse Video of Normal Mitosis in ESI-035-H2B-mCherry Cells, Related to Figure 1

Video S2. Time-Lapse Video of Lagging Chromosome during Mitosis in ESI-035-H2B-mCherry Cells, Related to Figure 1

**Figure 2:**
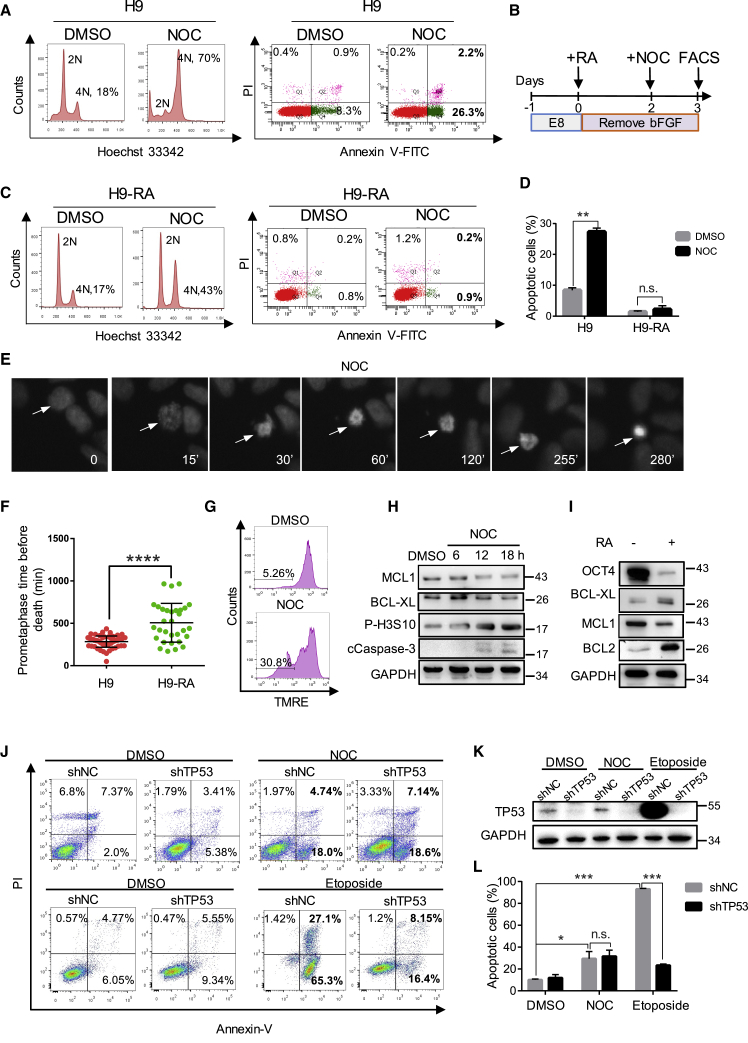
NOC Treatment Caused Rapid Apoptosis in hPSCs (A) H9 cells were treated with NOC, FACS analysis of cell-cycle profile, and apoptosis by Hoechst 33342 and Annexin V-PI staining. The control was DMSO-treated cells (n > 3). (B) Schematic timeline of the experiment/treatments. (C) Cell-cycle profile and apoptosis rate of NOC-treated differentiated (RA^+^) H9 analyzed by Hoechst and Annexin V-PI staining and FACS (n > 3 independent experiments). (D) Bar graph presentation of apoptosis rate in NOC-treated H9 cells (A) and differentiated H9 cells (C) Average apoptotic cell percentages are generated from three independent biological replicates (data presented as mean ± SEM; n.s., not significant; ^∗∗^p < 0.01, based on unpaired Student's t test). (E) Images of H2B-mCherry cells treated with NOC; the time (in minutes) is indicated at the bottom of each frame. The arrow indicates a cell that entered prometaphase at 15 min, arrested until 255 min, and died at 280 min. (F) Dot plot of the prometaphase duration of NOC-treated H9 cells (n = 53) and differentiated (RA^+^) H9 (n = 31) before cell death. Each dot represents one cell measured in the time-lapse video (data are presented as mean ± SEM; ^∗∗∗∗^p < 0.0001, based on unpaired Student's t test, data pooled from three independent experiments). (G) FACS analysis of mitochondrial membrane potential by TMRE dye staining during NOC treatment. The numbers represent the percentage of cells with reduced TMRE fluorescence (n > 3 independent experiments). (H) Western blot analysis of MCL1, BCL-XL, P-H3S10, cCaspase-3, and GAPDH protein levels (n = 3). (I) Western blot of OCT4, BCL-XL, MCL1, BCL2, and GAPDH protein expression in undifferentiated (RA^−^) and differentiated (RA^+^) hPSCs (n > 3 independent experiments). (J) FACS analysis of apoptosis in shNC and shTP53 H9 cells treated with NOC or etoposide by Annexin V-PI staining (n = 3 independent experiments). (K) Western blot showing knocking down of TP53 protein by shRNA. TP53 level was significantly upregulated by etoposide treatment but not NOC treatment in shNC cells. (L) Bar graph comparing the percentage of apoptotic cells in NOC- or etoposide-treated shNC and shTP53 H9 cells (data are presented as mean ± SEM of three independent experiments; n.s., not significant; ^∗^p < 0.05, ^∗∗∗^p < 0.001, based on unpaired Student's t test).

Video S3. Time-Lapse Video Showing Massive Cell Death of NOC-Treated, Prometaphase Arrested H9-H2B-mCherry Cells, Related to Figure 2

### Undifferentiated hPSCs Are Highly Sensitive to Abnormal Mitosis-Induced Apoptosis, which Is Independent of TP53

hPSCs have been reported to escape mitotic arrest due to uncoupling of the mitotic checkpoint from apoptosis (Mantel et al., 2007). However, they are highly sensitive to apoptosis upon DNA damage (Liu et al., 2013). To test whether hPSCs undergo apoptosis following perturbation of mitosis, we exposed hPSCs to nocodazole (NOC), which prevents the formation of the mitotic spindle. NOC treatment for 18 h caused cell-cycle arrest in G_2_/M, as indicated by the increase of 4N cells from 18% to 70% (Figure 2A). Strikingly, 29% of cells were found to undergo apoptosis with NOC treatment (Figure 2A). Similar results were obtained with human induced pluripotent stem cells (hiPSCs) (Figure S1A). In contrast to hPSCs, when mouse embryonic stem cells (mESCs) were treated with NOC, a significant proportion of cells slipped through mitotic arrest without division and formed tetraploid cells (8N) (Figure S1B), suggesting that there are major differences in the control of mitotic checkpoint between human and mouse PSCs.

We next examined the response of differentiated hPSCs toward NOC treatment. All-*trans* retinoic acid (RA) was used to induce differentiation (Figure 2B). After 1 day of NOC treatment, only 43% cells were arrested in G_2_/M, and less than 3% cells were apoptotic, much lower than that in undifferentiated hPSCs (29%) (Figures 2C and 2D). To exclude the possibility of 4N cells slipping into G_1_ phase, we monitored the human embryonic stem cell (hESC) division following NOC treatment by time-lapse imaging. As shown in Figure 2E, chromatin started to condense from 15 min and remained as metaphase chromosomes until 255 min. The arrested cells then died at about 280 min without exit from mitosis (Figure 2E). In contrast to undifferentiated H9 cells, RA-treated H9 cells stayed in metaphase arrest for about 480 min before entering apoptosis (Figure 2F). These results indicated that metaphase arrested hPSCs are much quicker to enter mitotic death than differentiated cells. We next investigated the role of mitochondrial integrity and TP53 during mitotic arrest-induced cell death of hPSCs. TMRE is a mitochondrial membrane potential indicator that displays strong red fluorescence when mitochondrial function is normal. Upon mitochondrial damage, the membrane potential decreases and the red fluorescence of TMRE diminishes accordingly. Fluorescence-activated cell sorting (FACS) results revealed that about 31% of hPSCs showed reduced TMRE signal after 18 h of NOC treatment, which was consistent with their apoptosis rate (Figures 2G and S1E). Western blot revealed significant upregulation of cleaved caspase-3 (cCaspase-3) and downregulation of anti-apoptotic protein BCL-XL and MCL1 in undifferentiated hESCs, which confirmed the initiation of apoptosis process (Figure 2H). In RA-treated H9 cells, the expression of the anti-apoptotic protein MCL1 decreased but BCL-XL and BCL-2 proteins increased markedly (Figure 2I), which may explain the reduced apoptosis of these cells after NOC treatment. To test whether mitotic death of hPSC is dependent on TP53, we used short hairpin RNA (shRNA) to knock down TP53 in H9 cells. Unexpectedly, TP53 knockdown did not decrease NOC-induced hPSC death, but dramatically alleviated cell death induced by etoposide, a DNA-damaging drug by inhibiting topoisomerase (Figures 2J and 2L). Western blot confirmed that TP53 protein was significantly reduced by shRNA (Figure 2K). Strikingly, etoposide treatment, but not NOC treatment, induced massive increase of TP53 protein in shNC cells (Figure 2K). These results suggested that TP53 is involved in DNA damage-induced apoptosis but not mitotic arrest-induced apoptosis. In summary, the rapid cell death observed during mitotic arrest was not caused by TP53 upregulation in hPSC but was due to high mitochondrial priming.

**Figure 3:**
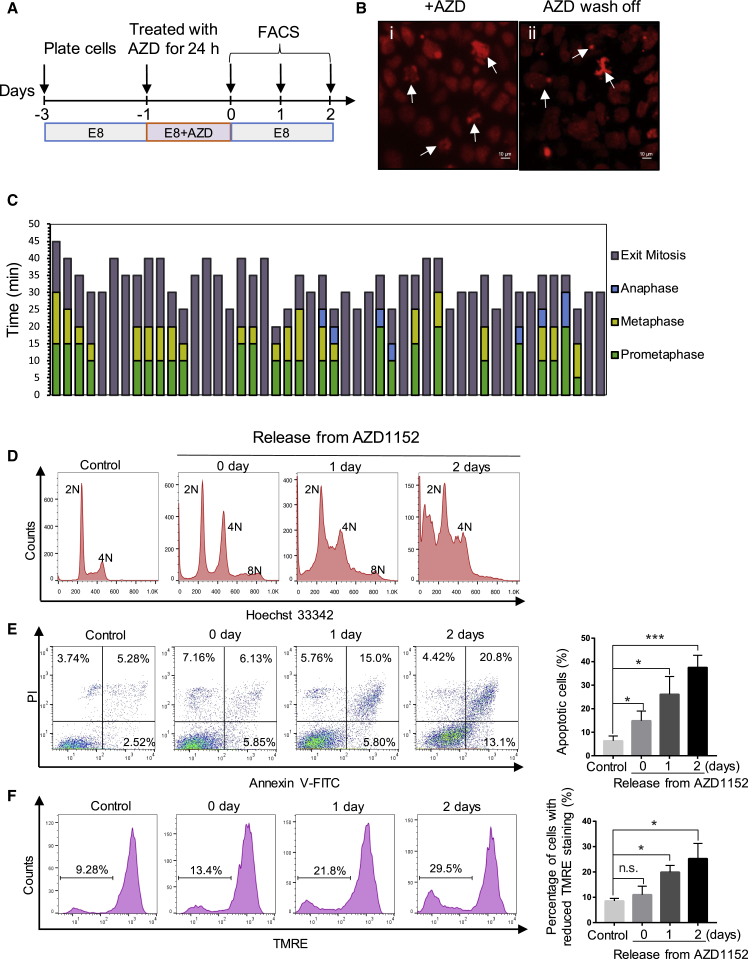
Abnormal Mitosis and Apoptosis Induced by Aurora Kinase B Inhibitor AZD1152 (A) Schematic timeline of the experiment. (B) Images of H2B-mCherry H9 cells treated with AZD1152 (AZD) and after AZD wash-off. Arrows indicate severely misaligned chromosomes. (C) Quantitative analysis of the length of each phase of the mitosis in AZD-treated cells (n = 48) based on live imaging. The prometaphase, metaphase, and anaphase are represented by different colors. Many cells condensed chromosomes but did not show distinguishable mitotic phases, thus represented as “Exit mitosis.” (D–F) Flow-cytometric analysis of cell cycle (Hoechst 33342, D), apoptosis (Annexin V-PI staining, E), and mitochondrial membrane potential (TMRE, F) at different time points after H9 cells were released from 24-h AZD treatment (error bars are shown as ±SEM of three independent biological experiments; n.s., not significant; ^∗^p < 0.05, ^∗∗∗^p < 0.001, based on unpaired Student's t test).

We next tested whether hPSCs initiate apoptosis after highly abnormal mitosis. Diploid human cells are known to be unstable in the tetraploid state, although they often form near-diploid aneuploid cells through multipolar division (Ganem et al., 2007). H9 cells expressing H2B-mCherry were treated with AZD1152, a specific inhibitor of Aurora B kinase, which has been shown to cause chromosome misalignment and cytokinesis failure (Sharif et al., 2010). Time-lapse imaging analysis showed that AZD1152 (AZD)-treated hPSCs had severely misaligned chromosomes during mitosis (Figure 3Bi). Many cells had condensed chromosomes, indicating that they have entered mitosis, but no discernible prometaphase, metaphase, or anaphase was observed (Figure 3C). Other cells decondensed chromosomes without clear separation of sister chromatids, thus making anaphase and telophase indistinguishable (Figure 3C). DNA content analysis showed that by 24 h, a significant proportion of cells became tetraploid (8N) (Figure 3D). After washing off AZD, the treated cells underwent multipolar divisions with severely misaligned chromosomes (Figure 3Bii) and their DNA content decreased to near-diploid levels (2N and 4N) in 2 days (Figure 3D). Two days after AZD removal, 34% of cells entered apoptosis and 30% cells had reduced TMRE fluorescence (Figures 3E and 3F). Together, these results suggest that following abnormal mitosis, hPSCs readily undergo apoptosis due to high mitochondrial priming, and this may be an important mechanism to eliminate stem cells that may gain chromosome abnormalities.

### NOXA Knockout or BCL-XL Overexpression Significantly Reduced Apoptosis of Cells Undergoing Erroneous Mitosis

**Figure 4:**
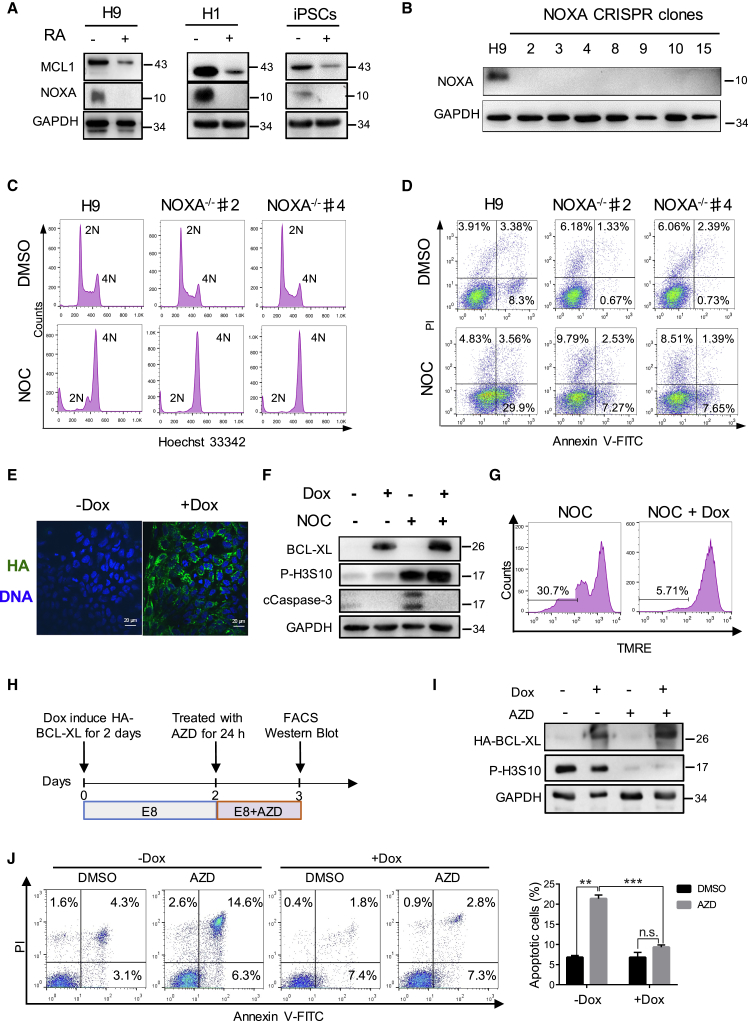
Knocking Out NOXA or BCL-XL Overexpression Suppressed Cell Death Caused by Abnormal Mitosis (A) Western blot showing NOXA protein is enriched in undifferentiated H9 cells, H1 cells, and hiPSCs, and significantly decreased upon RA-induced differentiation (+) (n = 3 independent experiments). (B) Western blot showing that seven NOXA^−/−^ clones had no NOXA protein expression. GAPDH was used as the loading control. (C) FACS analysis of cell-cycle profile by Hoechst 33342 staining of wild-type H9 and NOXA^−/−^ clones treated with NOC for 18 h (n > 3 independent experiments). (D) FACS analysis of apoptosis by Annexin V-PI staining of wild-type H9 and NOXA^−/−^ clones treated with NOC for 18 h (n > 3 independent experiments). (E) Immunostaining of HA-BCL-XL localization in H9 cells. HA-tag, green; DNA, blue. (F) Western blot analysis of ectopic BCL-XL, phosphorylated H3S10 (P-H3S10), and cCaspase-3 protein level. The different treatment combinations are indicated above the western blots (n = 3). (G) Flow-cytometric analysis of mitochondrial membrane potential by TMRE dye staining. Note that TMRE intensity did not drop in BCL-XL overexpression cells (+Dox) (n = 3 independent experiments). (H) Timeline of the Dox-inducible BCL-XL expression and AZD treatment. (I) Western blot analysis showing that Dox-induced BCL-XL overexpression and AZD abolished P-H3S10 (n = 3 independent experiments). (J) Flow-cytometric analysis of apoptosis (Annexin V-PI staining) of Dox-induced and non-induced H9 cells treated with AZD (data are presented as mean ± SEM of three independent experiments; n.s., not significant; ^∗∗^p < 0.01, ^∗∗∗^p < 0.001, based on unpaired Student's t test).

NOXA is a BH3 domain-only proapoptotic protein highly expressed in undifferentiated hPSCs and decreased dramatically upon RA-induced differentiation (Figure 4A). To test whether it is responsible for the rapid apoptosis of hPSC during abnormal mitosis, we used CRISPR to knock out *NOXA* gene in H9 cells. Four single guide RNAs (sgRNAs) were designed against the first and second exons of *NOXA* to ensure deletion of the coding region (Figure S2A). We picked 24 clones, 7 of which had a deletion in *NOXA* exons. Western blot confirmed the absence of NOXA protein in these clones (Figure 4B). We treated NOXA^−/−^ clones 2 and 4 with NOC and most cells became arrested in G_2_/M phase (4N) (Figure 4C), but there were no more than 10% of cells undergoing apoptosis in NOXA^−/−^ cells compared with 33% in wild-type H9 cells (Figures 4D and S2B). Thus, high levels of NOXA protein appeared to be an important contributing factor to the low apoptosis threshold in hPSCs.

Increased copy number of the chromosome 20q11.21 region can provide hPSCs with a survival advantage (Amps et al., 2011, Avery et al., 2013), and the *BCL2L1* gene product BCL-XL had been shown to be responsible for the anti-apoptotic effect of this amplicon (Avery et al., 2013). To test whether BCL-XL increases the survival of hPSCs treated with NOC, we generated doxycycline (Dox)-inducible BCL-XL hPSCs (Figure S2C). The addition of Dox led to the expression of hemagglutinin (HA)-tagged BCL-XL being detected by both immunostaining and western blot (Figures 4E and 4F). Induced expression of BCL-XL markedly reduced cell death caused by NOC treatment. The apoptosis rate was 4% with Dox induction versus 14% without Dox (Figures S2D and S2E). Dox-induced cells had significantly lower level of cCaspase 3 protein (Figure 4F), and maintained normal TMRE fluorescence intensity (plus Dox/minus Dox = 31%:6%) (Figure 4G). Similarly, BCL-XL overexpression also decreased the percentage of apoptosis caused by AZD treatment from 21% to 10% (Figures 4I and 4J). These results suggest that BCL-XL can reduce mitotic cell death by enhancing mitochondrial resilience.

To obtain a comprehensive assessment of how NOXA knockout or BCL-XL overexpression affect cells in normal conditions and under mitotic stress, we also performed global transcriptome profiling on five cell lines including wild-type, BCL-XL overexpression (BCL-XL), and NOXA^−/−^ H9 cells (Figure S4A). Principal component analysis showed that the transcriptome of wild-type, BCL-XL, and NOXA^−/−^ H9 cells were largely similar, but became significantly different upon NOC treatment (Figure S4B). Compared with control cells, NOC-treated BCL-XL and NOXA^−/−^ cells did not upregulate the NIK/NF-κB or apoptosis pathway gene, suggesting that the activation of these two pathways was inhibited by the anti-apoptotic mutations (Figures S4C and S4D; Tables S1 and S2). A similar trend was observed in iPSCs (Table S3). The pattern of global gene expression correlated with the resilient phenotype of BCL-XL and NOXA^−/−^ hESCs under mitotic stress.

### hPSCs with 20q11.21 CNV or BCL-XL Overexpression Inhibit Cell Death following Mitotic Stress and Permit Aneuploidy Formation

**Figure 5:**
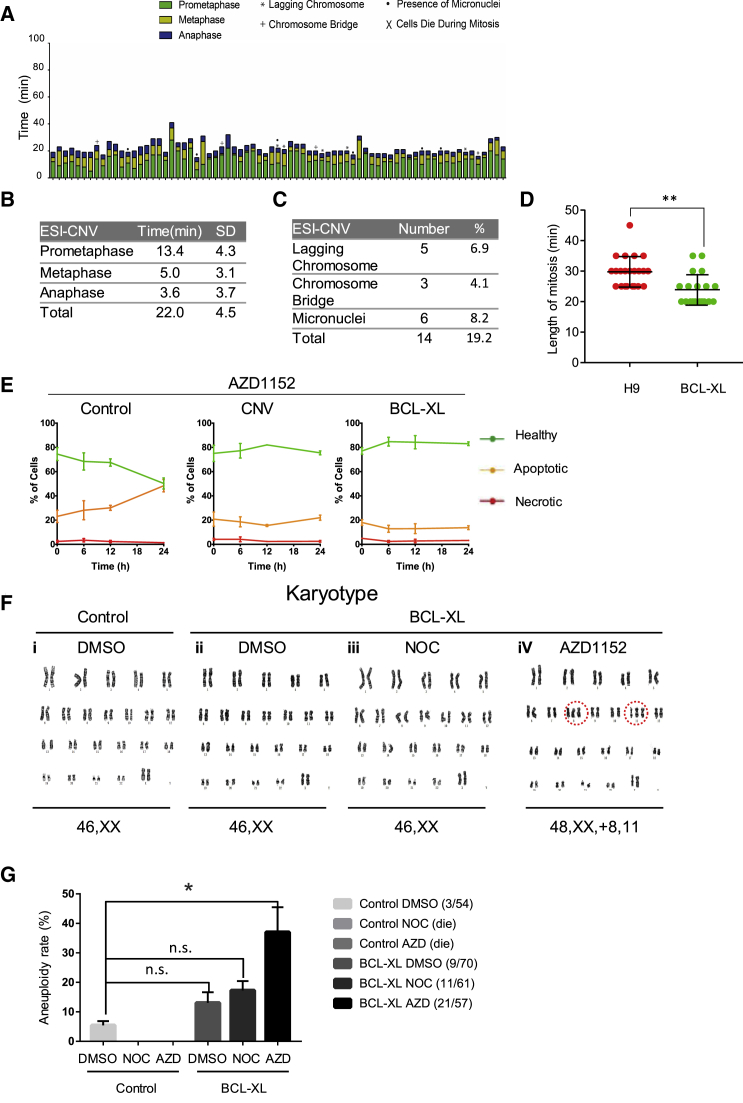
Altered Mitotic Kinetics, Mitochondrial Morphology, Cell Survival, and Tumorigenicity of ESI-CNV hPSCs and BCL-XL Overexpression Cells (A) Quantitative analysis of the length of each phase of the mitosis of ESI-CNV cells (n = 73, data pooled from more than three independent experiments) based on time-lapse movies. Prometaphase, metaphase, and anaphase are represented by different colors, and different types of division defects are marked by indicated symbols as in Figure 1. (B) Mean duration (minutes) of prometaphase, metaphase, and anaphase of ESI-CNV-H2B-mCherry cells. (C) Frequency of defective divisions in ESI-CNV-H2B-mCherry cells. (D) Dot plot of the mitosis duration in wild-type H9 (n = 23) and BCL-XL OE (n = 21) cells. Each dot represents one cell measured in the time-lapse video (data pooled from three independent experiments). Data are presented as mean ± SEM; ^∗∗^p < 0.01, based on unpaired Student's t test. (E) ESI-035 control, CNV, and BCL-XL OE cell lines were cultured for 24 h in the presence of 100 nM AZD. The levels of apoptosis were measured at 6, 12, and 24 h following inhibitor exposure. Solid lines indicate healthy cells (green), apoptotic cells (orange), and necrotic cells (red). Control cells displayed increased cell death following 24-h exposure when compared with CNV and BCL-XL OE cells. Error bars represent SEM from three biological replicates. (F) Karyotype analysis of DMSO-, NOC-, and AZD-treated BCL-XL OE cells. Red circle highlights trisomy of chromosomes 8 and 11. (G) Bar graph of aneuploid cell percentage in DMSO-, NOC-, and AZD-treated BCL-XL OE cells (values shown are mean ± SEM from three independent experiments; n.s., not significant; ^∗^p < 0.05, based on unpaired Student's t test).

Since increased BCL-XL expression suppresses cell death caused by NOC-induced prometaphase arrest, we studied whether hPSCs with an amplification of chromosome 20q11.21 were also more resilient to cell death following abnormal mitosis. ESI-CNV hPSC is a subline of ESI-035 hPSC with chromosome 20q11.21 CNV as the only mutation (Avery et al., 2013). We filmed mitotic events in the ESI-CNV cells that carry the 20q11.21 amplicon (Figure 5A). In these cells, prometaphase was 13.4 ± 4.3 min. The metaphase and anaphase durations were significantly shorter, 5.0 ± 3.1 min and 3.6 ± 3.7 min, respectively. The total division time was shorter in ESI-CNV-H2B-mCherry cells (22.0 ± 4.5 min) compared with that of the normal ESI-035 cell line (29.0 ± 10.3 min). Besides, BCL-XL OE cells also had shorter division time compared with wild-type H9 (Figure 5D). Aberrant mitoses were also seen in ESI-CNV cells (19%), compared with 33% in ESI-035 cells. We did not see cell death after abnormal mitosis in ESI-CNV cells (Figure 5A). Similar to inducible BCL-XL H9 cells, ESI-CNV and BCL-XL overexpression cells both showed a reduced rate of cell death after AZD treatment (Figure 5E). The level of BCL-XL overexpression was comparable with that in BCL2L1 CNV cells reported in other studies (Figure S5). Finally, as BCL-XL protected hPSCs from apoptosis following highly abnormal mitosis, we next tested whether it can help the survival of aneuploid cells. To this end, we treated Dox-induced or non-induced BCL-XL H9 cells with NOC or AZD for 18 h, by which time a significant proportion of cells had arrested in the prometaphase of cell cycle or had failed cytokinesis. The drugs were then washed off and cells were allowed to resume cell-cycle progression. After cells grew back, they were treated with NOC or AZD for another 18 h. No wild-type H9 cells grew back after repeated NOC or AZD treatment and release, whereas BCL-XL OE cells survived (Figure 5F). Karyotype analysis revealed that some AZD-treated and released BCL-XL OE cells had gained chromosome abnormality, one with extra chromosome 8 and 11 (Figure 5Fi–iv) and another one with trisomy 12 (data not shown). The aneuploidy rate increased to 37% in AZD-treated BCL-XL OE cells compared with that in control cells (Figure 5G). Even under normal conditions, BCL-XL overexpression increased the aneuploid rate from 6% to 12% on average, although this was not statistically significant (Figure 5G). Collectively, these results demonstrated that a low threshold of apoptosis is important to safeguard hPSCs from gaining chromosome abnormality after highly aberrant mitosis.

### Anti-apoptotic Mutations Enhance hPSC Survival and Lead to Larger Teratoma Formation

**Figure 6:**
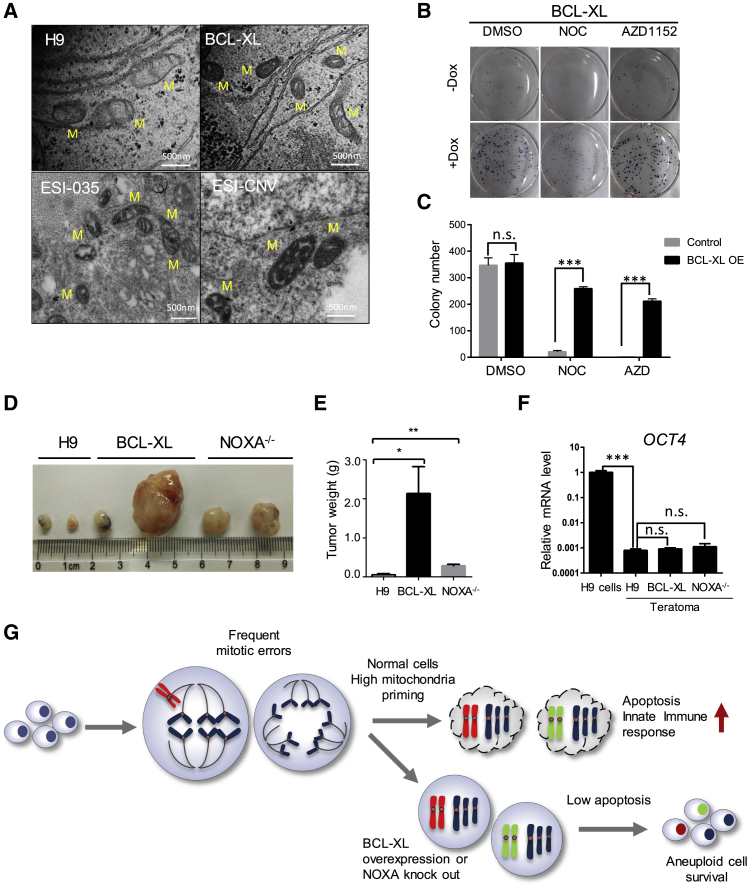
Altered Mitochondrial Morphology, Cell Survival, and Tumorigenicity of ESI-CNV hPSCs and BCL-XL Overexpression Cells (A) Transmission electron microscopy images of mitochondria in wild-type, BCL-XL H9 cells, and ESI-035, ESI-CNV cells. M, mitochondria. Scale bar, 500 nm. (B) Drug treatment and colony survival assay of Dox-inducible BCL-XL OE H9 cells. Cells were seeded at 5,000 cells/cm^2^, cultured for 7 days, and treated with DMSO, NOC, or AZD for 18 h. Thereafter, drugs were removed and cells were cultured for another 3 days followed by alkaline phosphatase assay (dark-blue color). Significantly more colonies survived in +Dox-induced groups (n = 3 independent experiments). (C) Bar graph representation of colony number of wild-type and BCL-XL OE cells treated with DMSO, NOC, or AZD for 18 h. After drug treatment, cells were cultured for 2 days and colonies were visualized by alkaline phosphatase assay (dark-blue color). Data are presented as mean ± SEM of three independent experiments; n.s., not significant; ^∗∗∗^p < 0.001, based on unpaired Student's t test. (D) Images of teratomas from wild-type, BCL-XL OE, and NOXA^−/−^ H9 cells. (E) Weight of teratomas formed by wild-type, BCL-XL OE, and NOXA knockout H9 cells (data pooled from n = 3 independent experiments). Data are presented as mean ± SEM; ^∗^p < 0.05, ^∗∗^p < 0.01, based on unpaired Student's t test). (F) qRT-PCR analysis of pluripotent genes (*OCT4*) in teratomas from wild-type, BCL-XL OE, and NOXA^−/−^ H9 cells (data from n = 3 independent experiments). Data are presented as mean ± SEM; ^∗∗∗^p < 0.001, based on unpaired Student's t test; n.s., not significant. (G) Cartoon illustrating how anti-apoptotic mutations may render hPSCs insensitive to mitotic stress and permit aneuploidy cell formation.

To better understand how BCL-XL protects mitochondria, we performed transmission electron microscopy (TEM) on hPSCs. Mitochondria in BCL-XL OE H9 or ESI-CNV cells had darker and more robust cristae compared with wild-type and ESI-035 cells (Figure 6A), suggesting mitochondrial membrane remodeling may have contributed to the enhanced survival of these cells. A number of genes that potentially may enhance mitochondrial function were also significantly upregulated in BCL-XL OE and NOXA^−/−^ H9 cells, such as APOO, TIMM10, NDUFB3, NDUFA9, MT-ATP6 and 8, MT-ND1, and MT-CYB (Figure S6A). We next tested the clonogenicity and tumorigenicity of hPSCs with increased levels of BCL-XL. Inducible BCL-XL cells formed more colonies when plated as single cells at low density after Dox addition. Similarly, more clones survived after NOC or AZD treatment in Dox-induced cells (Figures 6B and 6C). We transplanted 1 × 10^6^ cells to the groin position of immune-deficient mice. After 8 weeks, BCL-XL OE H9 cells formed larger teratomas compared with wild-type H9 cells. NOXA^−/−^ H9 cells also formed bigger teratomas, although not as large as BCL-XL H9 cells (Figures 6D and 6E). To determine whether these teratomas contain undifferentiated cells, we examined the expression level of pluripotent genes *OCT4*. Teratomas from wild-type, BCL-XL, and NOXA^−/−^ H9 also downregulated *OCT4* mRNA level, implying the absence of undifferentiated cells (Figure 6F). Histological analysis of the teratomas showed that wild-type, BCL-XL, and NOXA^−/−^ H9 cells all had well-differentiated ectoderm, mesoderm, and endoderm tissues (Figure S6B). In addition, BCL-XL OE or NOXA^−/−^ cells all downregulated *OCT4* and upregulated *SOX17* and *FOXA2*, similar to wild-type H9 cells during *in vitro* endoderm differentiation (Figures S6C and S6D). Together, these results indicated that these anti-apoptotic mutations promoted hPSC survival and teratoma growth but did not affect their differentiation potential.

## Discussion

In this study we have shown that hPSCs exhibit a relatively high frequency (30%) of division errors (measured through time-lapse imaging), as evidenced by the presence of lagging chromosomes, chromosome bridges, micronuclei, and, to a lesser extent, multipolar divisions. As hPSCs can self-renew indefinitely, the likelihood of gaining chromosomal abnormality increases substantially during long-term *in vitro* culture. It has been reported that the mitotic checkpoint is uncoupled from apoptosis in hPSCs (Mantel et al., 2007). However, we found that hPSCs were not able to slip into the next phase of the cell cycle during NOC treatment. Instead, we found a rapid drop in their mitochondrial membrane potential and cell death within about 5 h post treatment, suggesting that the mitotic checkpoint is functional in hPSCs and that the high mitochondrial priming in undifferentiated hPSCs ensures rapid death of cells experiencing mitotic stress. This is in stark contrast to the response of somatic cells and mESCs. For example, MCF7 human breast cancer cells and RPE1 cells start to die or slip into interphase after about 10 h of prometaphase arrest (Brito and Rieder, 2006, Orth et al., 2012, Uetake and Sluder, 2010). Similarly, shortly after differentiation, hPSCs could also withstand longer prometaphase arrest (close to 10 h) before slipping to anaphase or entering the next cell cycle without division. Meanwhile, genome-damaged cells would enter senescence and then die slowly like many somatic cells (Bai et al., 2017, di Fagagna, 2008).

It was notable that NOC treatment did not induce significant TP53 upregulation in hPSCs, whereas the DNA damage-inducing drug etoposide led to a substantial induction of TP53. Two groups reported that in hESCs, rapid apoptosis occurred after DNA damage in a TP53-dependent manner (Liu et al., 2013, Dumitru et al., 2012). In our experiments, TP53 knockdown did not protect cells from NOC treatment-induced cell death but alleviated etoposide-induced cell death (Figures 2J–2L), suggesting that different mechanisms underlie cell death induced by abnormal mitosis and DNA damage, albeit both involve mitochondria. As NOC-induced apoptosis is TP53 independent, cells with *BCL2L1* amplification will have a survival advantage in the event of abnormal mitosis. Indeed, we found that the proportion of aneuploid cells increased from 6% to 12% on average after BCL-XL overexpression in hPSCs. Furthermore, BCL-XL OE cells exhibited 37% aneuploid rate after AZD treatment, whereas no wild-type cell survived repeated NOC or AZD treatment. In agreement with this result, the ISCI study found that, among five cell lines that gained the *BCL2L1* CNV at early passage, three had karyotypically abnormal cells in later culture (Amps et al., 2011). Together, these data suggest that anti-apoptotic mutations can render hPSCs insensitive to mitotic stress and enable the retention of cells with chromosomal abnormalities in culture.

The proapoptotic Bcl2 family member NOXA appears to be a rate-limiting factor in mitotic death. It is induced by G_2_/M arrest and can promote MCL1 degradation to accelerate apoptosis (Haschka et al., 2015). We found that NOXA is highly expressed in hPSCs, but quickly downregulated upon differentiation (Figure 4A). Furthermore, knocking out NOXA in hPSCs led to significantly less cell death during NOC treatment (Figure 4D). Therefore, we speculate that hPSCs may control NOXA expression to set a highly sensitive mitochondria-apoptosis threshold and rapidly eliminate cells going through abnormal mitosis.

Our transcriptome profiling found that anti-apoptotic mutations can also affect global gene expression, particularly when cells are under stress. Upon NOC treatment, wild-type H9 cells and iPSCs, but not BCL-XL OE and NOXA^−/−^ cells, upregulated both apoptosis and NIK/NF-κB pathway genes (Figures S4D–S4F). The NIK/NF-κB pathway could be activated by cytoplasmic DNA generated from different sources, such as micronuclei formed after chromosome mis-segregation or DNA damage (Crasta et al., 2012, Harding et al., 2017, Mackenzie et al., 2017), or mitochondria DNA leakage (Ichim et al., 2015, West et al., 2015). This is in accordance with our results that the mitochondrial membrane potential was much better maintained in cells with anti-apoptotic mutations. Therefore, anti-apoptotic mutations may prevent the activation of innate immune response and NIK/NF-κB pathway gene expression by strengthening mitochondria membrane.

The structure of the mitochondria is very different in pluripotent cells and differentiated cells. The inner cell mass (ICM) cells of human preimplantation embryos have been shown to have spherical mitochondria with very few cristae (Sathananthan and Trounson, 2000). Similar to ICM cells, hPSCs also have immature mitochondria with round and cristae-poor morphology (Folmes et al., 2012). Using TEM, we found that our wild-type hPSCs had cristae-poor mitochondria as described by other groups. In *BCL2L1* CNV cells and BCL-XL OE cells, mitochondria had darker and thicker cristae (Figure 6A). Anti-apoptotic members of Bcl2 family proteins have been shown to enhance mitochondrial integrity in multiple ways. For example, BCL-XL overexpression inhibits Ca^2+^-mediated multiple conductance channel opening to block mitochondrial membrane permeabilization (Tornero et al., 2011). Several studies reported that BCL-XL may also function as anti-oxidant with BCL-XL overexpression significantly reducing the levels of reactive oxygen species induced by GSK2578215A (Pfeiffer et al., 2017, Saez-Atienzar et al., 2016). These mechanisms may underlie the changed morphology and enhanced resilience of mitochondria observed in our study.

Despite the suppression of apoptosis, BCL-XL overexpression or NOXA knockout alone did not appear to lead to malignant transformation of hPSC. Transplanted undifferentiated BCL-XL OE and NOXA^−/−^ cells formed fully differentiated teratomas with well-defined borders that no longer contained pluripotent cells, albeit with significantly larger size (Figures 6D–6F). This suggests that anti-apoptotic mutations by themselves are not directly oncogenic but can help the survival of hPSCs and their differentiated derivatives. However, as elevated BCL-XL levels permitted cells with chromosomal abnormality to evade death, we think that constitutive overexpression of anti-apoptotic genes should be avoided if the differentiated progeny cells are to be used for transplantation. In our transcriptome study, we also identified several genes commonly upregulated in BCL-XL OE, NOXA^−/−^, and shTP53 cells (Figure S4I). Among these, Arrestin B1 (*ARRB1*) has been shown to positively regulate ERK1/2 and AKT signaling, while attenuating the NF-κB pathway, and so could enhance cell survival (Wang et al., 2006, Zhang et al., 2017). A number of long non-coding RNAs also increased several-fold in BCL-XL OE, NOXA^−/−^, and shTP53 cells. For example, LNC00458 (also aligned to BC026300) has been shown to have NANOG binding sites at its promoter and was only expressed in undifferentiated hESCs (Ng et al., 2012). Its expression level was 3- to 5-fold higher in BCL-XL OE, NOXA^−/−^, and shTP53 H9 cells compared with wild-type H9, H1, and hiPSCs. Taken together, genes identified in our study that were enriched in hPSCs with reduced apoptosis potential may be used as biomarkers to evaluate whether cells are predisposed to culture adaptation.

Our results have revealed the unique characteristics and regulatory mechanism of hPSC mitosis and apoptosis. In summary (Figure 6G), hPSCs express high levels of NOXA, which determines the high priming state of mitochondria. Although the error-prone mitosis in hPSCs can lead to the formation of cells with chromosome aberrations, they are quickly eliminated due to the low apoptosis threshold. BCL-XL overexpression or NOXA downregulation can desensitize cells to mitotic stress and enable aneuploidy formation.

To ensure the safety of hPSCs, methods to identify 20q11.21 CNV at single-cell level in an hPSC culture at relatively low cost are urgently needed. Sensitive and reliable biomarkers, including surface markers and the expression of certain coding or non-coding genes that have strong correlation with 20q11.21 CNV, will also be very useful for detection purposes. With the decrease in cost and increased throughput of second-generation DNA-sequencing technology, it will be possible to use sequencing-based assays to simultaneously analyze the transcriptome and the copy number of single-cell or single-cell-derived small colonies. As hPSCs are highly primed for apoptosis and the *BCL2L1* locus is frequently amplified in hPSCs, they provide a model to dissect the mechanism driving this adaptive mutation. Analyzing the changes in epigenetic modification and 3D genome around *BCL2L1* locus when hPSCs are under stress may provide important clues and shed light on how to better safeguard the genome integrity of stem cells.

## Experimental Procedures

The teratoma analysis was conducted in accordance with the Guide for the Care and Use of Animals for Research Purposes. The protocol for the teratoma formation assay in severe combined immunodeficiency mice was approved by Institutional Animal Care and Use Committee and Internal Review Board of Tsinghua University.

### hPSC Culture

The hPSC lines used in this study were ESC lines H9, H1 (WiCell Institute, Madison, WI, USA), human iPSC (generated from CD34^+^ human cord blood cells), and ESI-035 and ESI-035-CNV (A-STAR, Singapore) (Avery et al., 2013). The cells were maintained on inactivated mouse embryonic fibroblast (MEF) cells in culture medium consisting of KO-DMEM (Invitrogen) supplemented with nonessential amino acids (NEAA) (Invitrogen), 0.1 mM 2-mercaptoethanol (Sigma-Aldrich), 1 mM GlutaMAX (Invitrogen), 20% Knock-Out Serum-Replacement (Invitrogen), and 4 ng/mL basic fibroblast growth factor (Peprotech). Cells were cultured at 37°C in a humidified atmosphere of 5% CO_2_ in air. Cells were passaged with 1 mg/mL collagenase IV (Invitrogen) and seeded onto a 25-cm^2^ flask on feeder cells (mitomycin C-inactivated MEFs) For feeder-free culture, hPSCs were grown for more than three passages in the absence of feeders in E8 or in mTeSR (STEMCELL Technologies) on Matrigel (BD Bioscience) (all cells were regularly checked and confirmed free of mycoplasma contamination, and have diploid karyotype).

### Live-Imaging Experiments

Live-imaging experiments were performed on a Leica AF6000 inverted microscope with a 40× objective for H9-H2B-mCherry cells, and a Nikon TiE inverted fluorescence microscope fitted with an sCMOS camera using a 100× oil objective lens for ESI-035-H2B-mCherry and ESI-CNV-H2B-mCherry cells.

### Flow-Cytometry Analysis, Immunostaining, and Western Blot

Cell-cycle profile, apoptosis, and mitochondrial membrane potential were analyzed by Hoechst 33342 (Dojindo) staining, Annexin V/Dead Cell Apoptosis Kit (Invitrogen), and TMRE (Invitrogen) staining, followed by flow-cytometric analysis on a BD LSRFortessa FACS machine (BD Biosciences). The detailed immunostaining and western blot procedure, and antibodies used in this study can be found in Supplemental Information.

### Inhibitor Treatment

Nocodazole (Sigma) and AZD1152 (Tocris) were used to perturb mitosis in hPSC. H9, ESI-035, and ESI-CNV cells were treated 50 ng/mL NOC or 100 nM AZD1152 for appropriate durations as specified in each experiment. Etoposide was used to induce DNA damage in hPSC. H9 cells were treated with 20 nM etoposide (TargetMol) for 5 h.

### Generation of H2B-mCherry, BCL-XL Overexpression, TP53 Knockdown, and NOXA Knockout Cell Lines

H2B-mCherry ESI-035, H2B-mCherry ESI-035-CNV, and Dox-inducible BCL-XL overexpression H9 lines were generated by transfection with pCAG-H2B-mCherry-IRES-puro plasmid, piggyBac plasmids with TRE3G promoter driving BCL-XL, and CAG promoter driving TetON-3G transactivator, respectively. TP53 knockdown H9 cells were constructed by infecting cells with shTP53 expressing lentivirus. NOXA knockout H9 cells were generated by Cas9 and sgRNA targeting TP53 gene. The detailed procedure is described in Supplemental Information.

### Clonogenicity and Karyotype Analysis

Dox-inducible BCL-XL H9 cells were seeded at 5,000 cells/cm^2^ and cultured for 7 days. Dox was added from day 0 onward. Cells were then treated with DMSO, NOC, or AZD for 18 h on day 4. After drugs wash-off, cells were cultured in hESC medium with or without Dox for 3 days. To quantify the number of surviving colonies, we fixed cells in 2% paraformaldehyde for 2 min, and revealed alkaline phosphatase activity by a mixture of NBT and BCIP (Roche) staining. For detection of aneuploidy cells, drug-paused cells were cultured until confluent and sent for karyotyping at the Reproductive Medicine Center of the Peking University Third Hospital.

### Transmission Electron Microscopy

For TEM study, H9 or ESI-035 cells were prefixed for 1 h at room temperature in 1.25% glutaraldehyde in hESC medium followed by fixation in 2.5% glutaraldehyde overnight at 4°C. Sample embedding and sectioning was performed by the TEM facility at Tsinghua University and the University of Sheffield. The TEM images were acquired using a Hitachi H-7650 transmission electron microscope at 80 KV.

### Statistics

All error bars represent standard error of the mean (SEM) unless otherwise indicated. An unpaired Student's t test was used to determine significance of data unless otherwise indicated.

## Accession Numbers

The RNA high-throughput sequencing data are publicly available at the National Center for Biotechnology Information with Gene Expression Omnibus (GEO), accession number GEO: GSE109786.

## Author Contributions

J.Z., A.J.H., I.B., and J.N. conceived the study and designed experiments; J.Z. and A.J.H. performed live-imaging experiments on H9 and ESI hPSCs; J.Z. performed mitosis and apoptosis studies in wild-type, BCL-XL OE, TP53 knockdown, NOXA^−/−^ H9, and human iPSCs; P.W. performed mitosis and apoptosis studies in mESCs; F.D. generated iPSCs and performed teratoma analysis; L.B. constructed piggyBac-based Dox-inducible overexpression system; H.Q. and R.H. performed RNA-sequencing data processing; F.Z. performed hPSC culture; D.R. and M.J. performed ESI hESC lines culture and FACS analysis; L.L. and P.J. generated shTP53 and shNC lentivirus; P.W.A. provided advice for the experiments and manuscript; J.Z., I.B., and J.N. wrote the manuscript.

## Supplemental Information

Document S1. Supplemental Experimental Procedures, Figures S1–S6

Table S1. GO Terms of BCL-XL Overexpression Affected Genes, Related to Figure S4

Table S2. GO Analysis of NOC Upregulated Genes in Wild-Type, BCL-XL OE, or NOXA Knockout H9 Cells, Related to Figure S4

Table S3. GO Analysis of NOC Upregulated Genes in Wild-Type Cells or BCL-XL OE iPSCs, Related to Figure S4

Document S2. Article plus Supplemental Information
