# Supplementary material for: Anti-apoptotic Mutations Desensitize Human Pluripotent Stem Cells to Mitotic Stress and Enable Aneuploid Cell Survival
Source: Stem Cell Reports. 2019 Feb 14;12(3):557–71. doi: 10.1016/j.stemcr.2019.01.013 (PMC6411485; doi:10.1016/j.stemcr.2019.01.013)
Supplement: Document S1. Supplemental Experimental Procedures, Figures S1–S6 [file mmc1.pdf]

Stem Cell Reports, Volume *12*

Supplemental Information

Anti-apoptotic Mutations Desensitize Human Pluripotent Stem Cells to Mitotic Stress and Enable Aneuploid Cell Survival

Jing Zhang, Adam J. Hirst, Fuyu Duan, Hui Qiu, Rujin Huang, Ying Ji, Lufeng Bai, Fengzhi Zhang, Darren Robinson, Mark Jones, Le Li, Peizhe Wang, Peng Jiang, Peter W. Andrews, Ivana Barbaric, and Jie Na

Figure S1

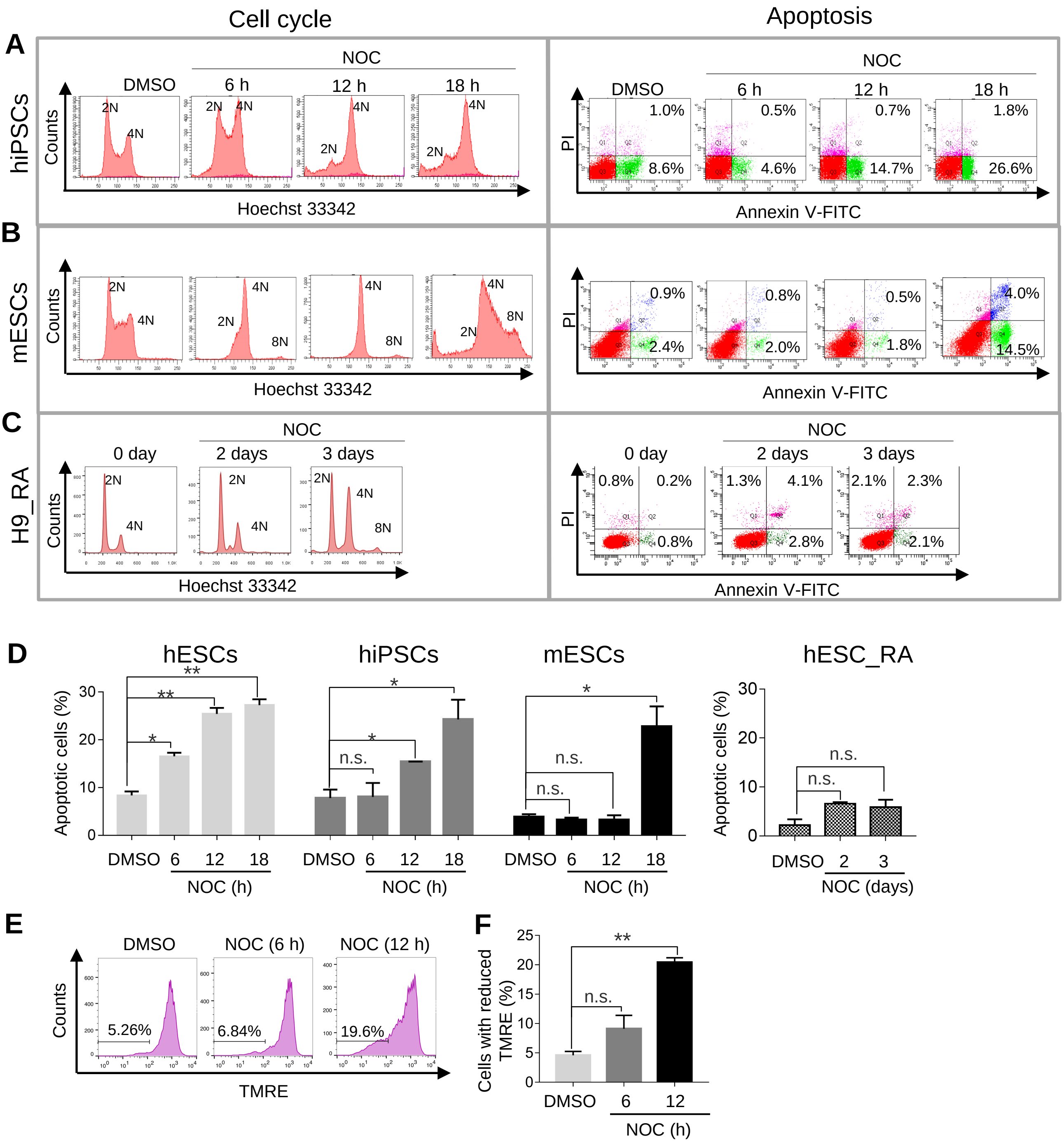

Figure S1. NOC treatment induced rapid apoptosis in hPSCs while mESCs and differentisted hPSCs can slip into the next phase of cell cycle in the presence of NOC, Related to Figure 2.

(A, B and C) FACS analysis of cell cycle profile (Hoechst staining) and apoptosis (Annexin V-PI staining) at different time points in NOC treated hiPSCs (A), mESCs (B) and differentiated H9 (RA+) cells (C)(n>3).

(D) Statistical analysis of apoptotic cell percentage in NOC treated hESCs (H9), hiPSCs, mESCs and differentiated H9 (RA+) cells. Average apoptotic cell percentage are generated from 3 independent biological replicates (Data are represented as mean ± s.e.m., n.s., not significant, *P < 0.05, **P<0.01, based on unpaired Student's *t*-test).

(E) FACS analysis of mitochondrial membrane potential by TMRE dye staining at indicated time points during NOC treatment. The numbers represents the percentage of cells with reduced TMRE fluorescence (n>3).

(F) Statistical analysis of H9 cells with reduced TMRE (E) after NOC treatment (Data are represented as mean s.e.m. of 3 biological replicates, n.s., not significant, **P<0.01, based on unpaired Student's *t*-test).

Figure S2

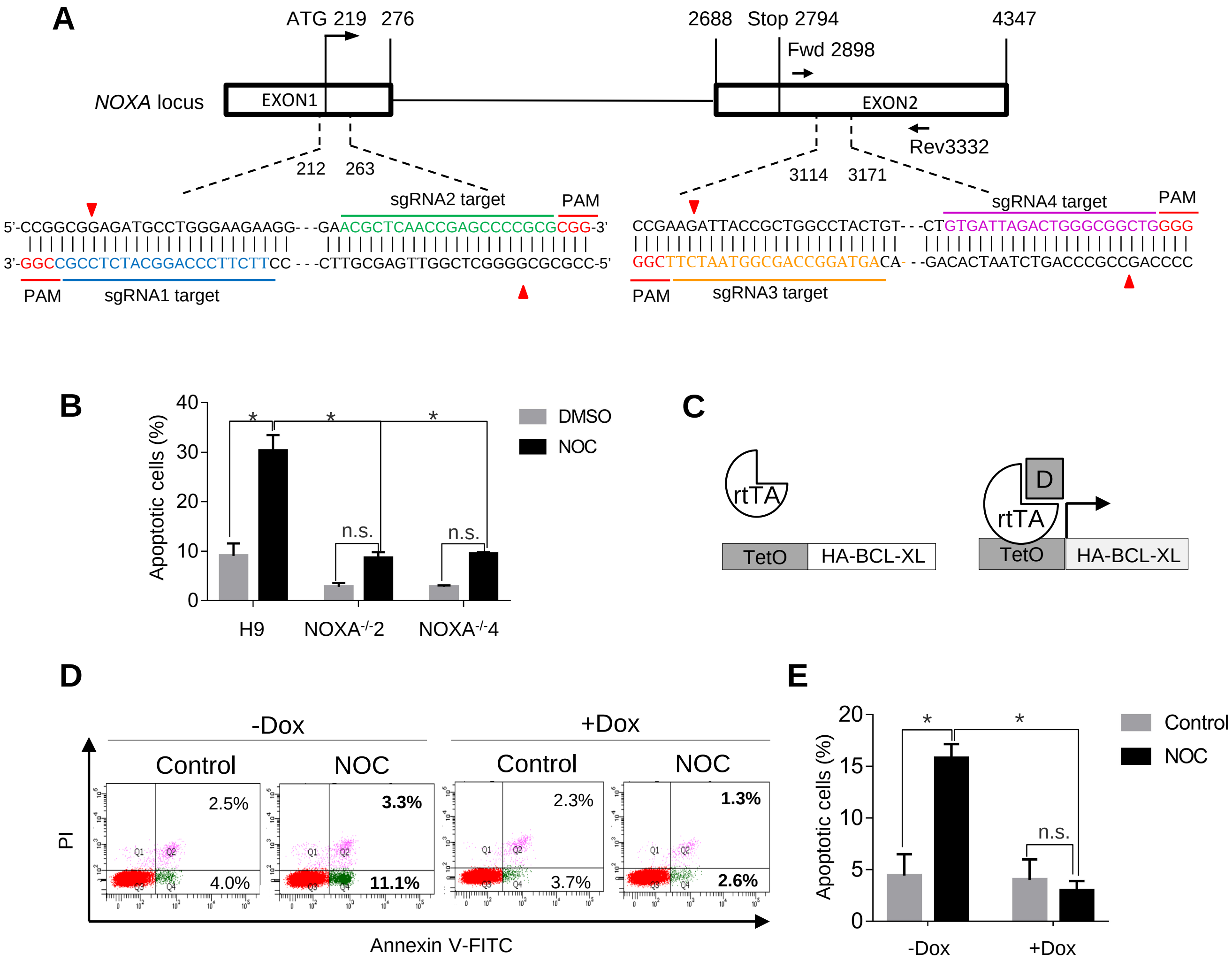

Figure S2. NOXA knock-out or BCL-XL overexpression decreased mitotic error induced apoptosis, Related to Figure 4.

(A) Schematic view of NOXA genome locus, the positions and sequence of crRNAs.

(B) Statistical analysis of apoptotic cell percentage in NOC treated wild-type H9 and NOXA$^{-/-}$ clones (Data are represented as mean ± s.e.m. of 3 biological replicates, n.s., not significant, **P* < 0.05, based on unpaired Student's t-test).

(C) Cartoon depicting the Dox-inducible BCL-XL expression system.

(D) Flow cytometric analysis of apoptosis (Annexin V-PI staining) of Dox-induced and non-induced HA-BCL-XL cells treated with NOC (n>3).

(E) Statistical analysis of apoptotic cell percentage from inducible HA-BCL-XL cells (D) with NOC treatment (Error bars are shown as ± s.e.m. of 3 independent biological experiments, n.s., not significant, **P* < 0.05, based on unpaired Student's t-test).

Figure S3

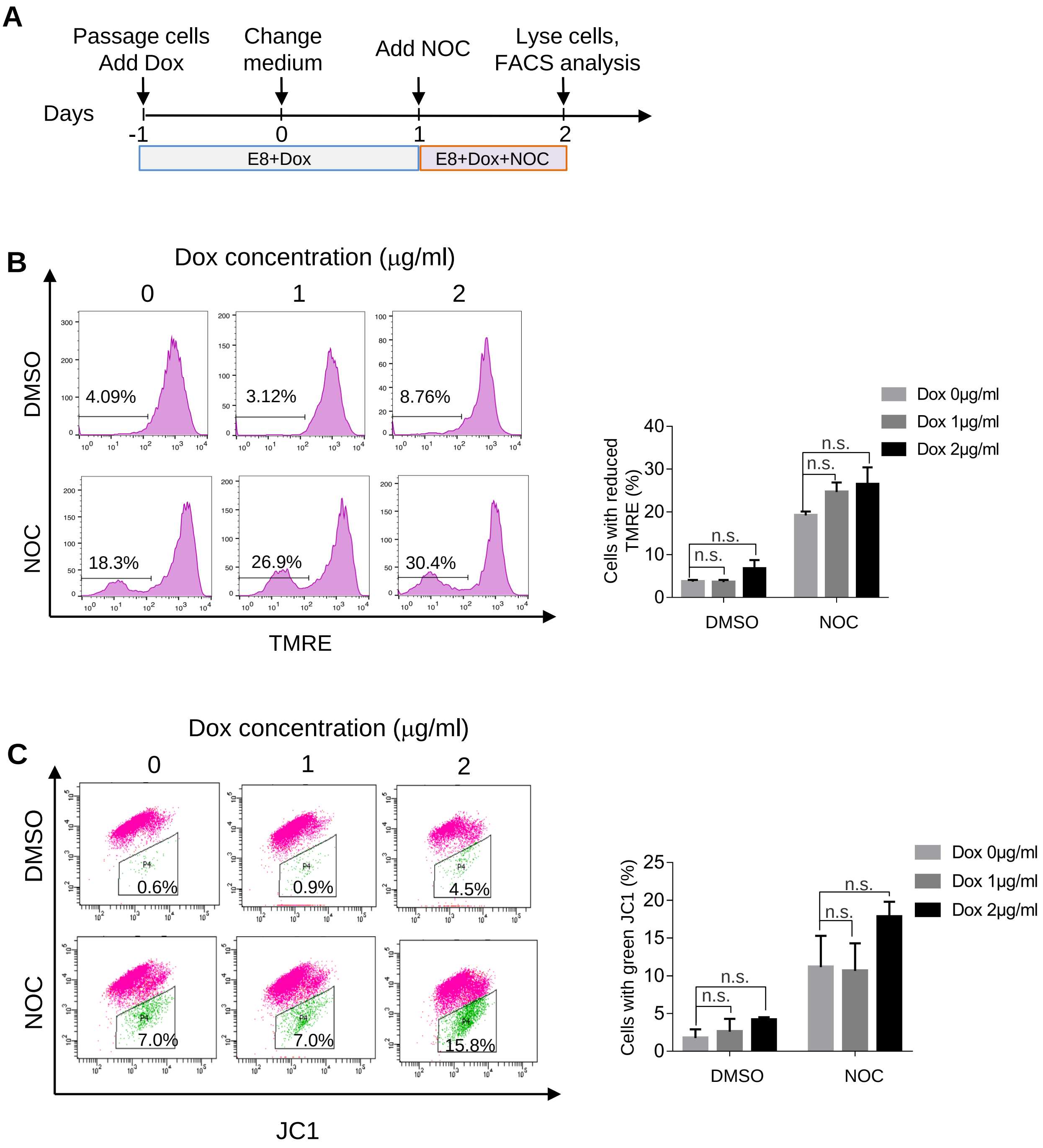

Figure S3. Dox alone does not affect apoptosis or mitochondrial membrane potential change induced by NOC treatment in H9 cells, Related to Figure 4.

(A) Timeline of the experiment. Dox was added right after passaging of cells, medium (with Dox) was changed 1 day after passaging. After another day, NOC was added for 24 h, followed by FACS analysis.

(B and C) Mitochondrial membrane potential was examined by TMRE and JC1 staining and FACS (n=3). The concentration of Dox was labeled on the top. (Data are represented as mean ± s.e.m. of 3 independent experiments, n.s., not significant, based on unpaired Student's *t*-test).

Figure S4

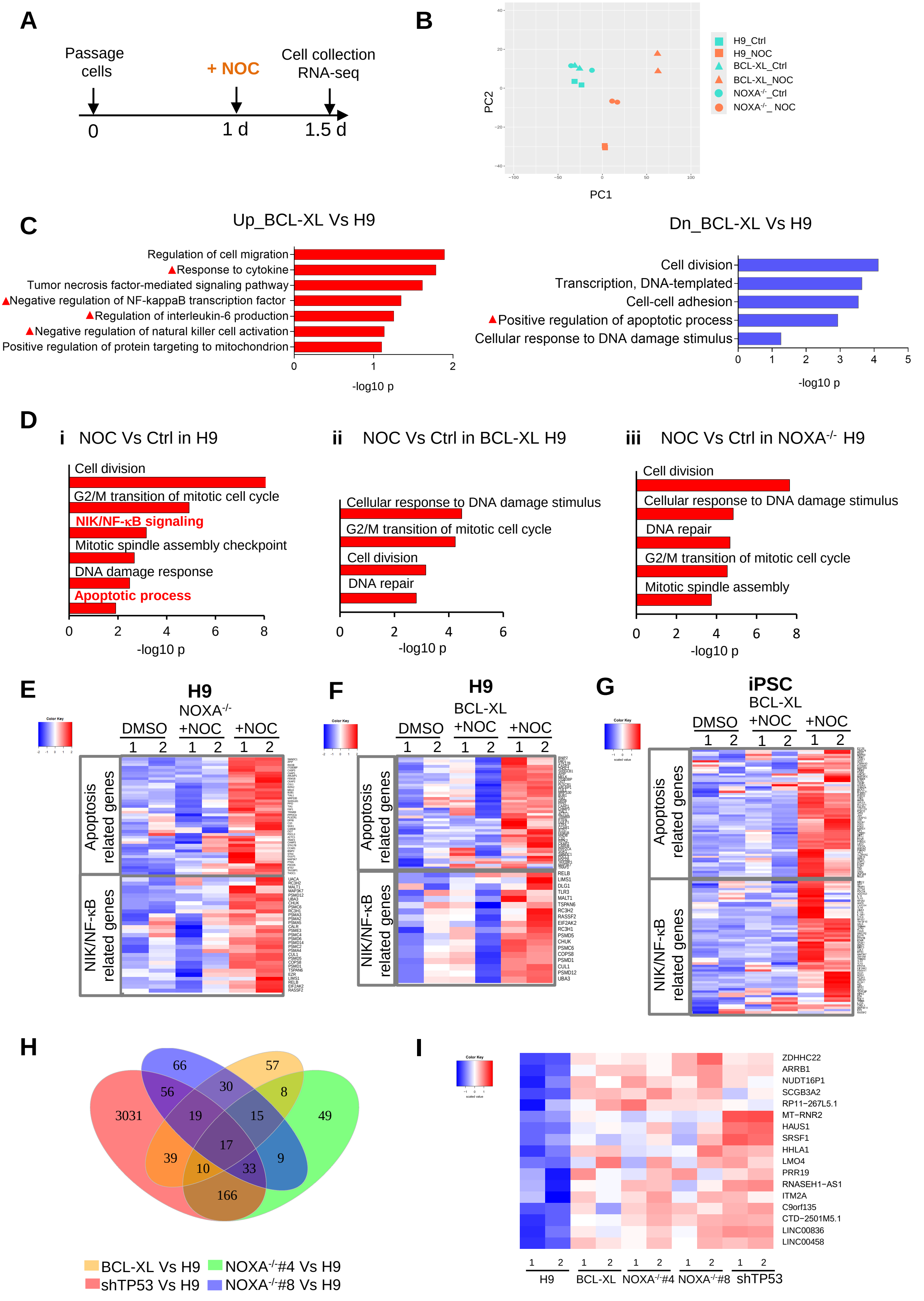

A
Passage cells
+ NOC
Cell collection RNA-seq
0
1 d
1.5 d
B
PC2
PC1
H9_Ctrl
H9_NOC
BCL-XL_Ctrl
BCL-XL_NOC
NOXA-/-_Ctrl
NOXA-/-_ NOC
C
Up_BCL-XL Vs H9
Regulation of cell migration
Response to cytokine
Tumor necrosis factor-mediated signaling pathway
Negative regulation of NF-kappaB transcription factor
Regulation of interleukin-6 production
Negative regulation of natural killer cell activation
Positive regulation of protein targeting to mitochondrion
-log10 p
Dn_BCL-XL Vs H9
Cell division
Transcription, DNA-templated
Cell-cell adhesion
Positive regulation of apoptotic process
Cellular response to DNA damage stimulus
-log10 p
D
i NOC Vs Ctrl in H9
Cell division
G2/M transition of mitotic cell cycle
NIK/NF-κB signaling
Mitotic spindle assembly checkpoint
DNA damage response
Apoptotic process
-log10 p
ii NOC Vs Ctrl in BCL-XL H9
Cellular response to DNA damage stimulus
G2/M transition of mitotic cell cycle
Cell division
DNA repair
-log10 p
iii NOC Vs Ctrl in NOXA-/- H9
Cell division
Cellular response to DNA damage stimulus
DNA repair
G2/M transition of mitotic cell cycle
Mitotic spindle assembly
-log10 p
E
H9
NOXA-/-
DMSO
+NOC
+NOC
Apoptosis related genes
NIK/NF-κB related genes
F
H9
BCL-XL
DMSO
+NOC
+NOC
Apoptosis related genes
NIK/NF-κB related genes
RELB
LIMS1
DLG1
TLR3
MALT1
TSPAN6
RC3H2
RASSF2
EIF2AK2
RC3H1
PSMD5
CHUK
PSMC6
COPS8
PSMD1
CUL1
PSMD12
UBA3
G
iPSC
BCL-XL
DMSO
+NOC
+NOC
Apoptosis related genes
NIK/NF-κB related genes
H
3031
66
57
49
56
30
8
19
15
17
39
9
10
33
166
BCL-XL Vs H9
NOXA-/-#4 Vs H9
shTP53 Vs H9
NOXA-/-#8 Vs H9
I
Color Key
ZDHHC22
ARRB1
NUDT16P1
SCGB3A2
RP11−267L5.1
MT−RNR2
HAUS1
SRSF1
HHLA1
LMO4
PRR19
RNASEH1−AS1
ITM2A
C9orf135
CTD−2501M5.1
LINC00836
LINC00458
H9
BCL-XL
NOXA-/-#4
NOXA-/-#8
shTP53

Figure S4. Transcriptome analysis of hPSCs with anti-apoptotic mutation after NOC treatment, Related to Figure 4.

(A) Schematic timeline of RNA-seq samples collection.

(B) Principal component analysis of different cell lines treated with NOC. Ctrl (DMSO treatment) in blue. NOC treatment in orange. Different shapes represent assigned cell lines. Two biological replicates for each group.

(C) Gene ontology (GO) analysis of differentially expressed genes in BCL-XL overexpression (BCL-XL) cells versus wild-type H9 cells. Red and blue represent GO term of genes significantly upregulated and downregulated in BCL-XL cells. Note that genes negatively regulating NF-kB transcription factor were higher expressed in BCL-XL cells. While genes positively regulating apoptotic processes were higher in wild-type H9 cells.

(D) Enriched GO terms associated with NOC treatment in different cell lines, wild-type H9 (i), BCL-XL H9 (ii), $NOXA^{-/-}$ H9 (iii).

(E, F and G) Heatmap of apoptosis and NIK/NF-κB pathway genes that are significantly differentially expressed in NOC treated wild-type H9 versus $NOXA^{-/-}$ H9 (E), NOC treated wild-type H9 versus BCL-XL H9 (F), NOC treated wild-type iPSC versus BCL-XL OE iPSC (G), respectively. Many apoptosis and NIK/NF-κB related genes were downregulated by BCL-XL overexpression or NOXA knockout. FPKM values were scaled and color key indicated relative gene expression level. Higher level in red, lower level in blue (n=2).

(H) Venn diagram showing intersect gene numbers between significantly upregulated genes when BCL-XL, $NOXA^{-/-}$ or shTP53 H9 were compared with wild-type H9.

(I) Heatmap of the 17 commonly upregulated genes from Venn diagram. These genes, such as ARRB1 and LNC00458, may be potential biomarkers to identify culture adapted cells. FPKM values were scaled and color key indicated gene expression level. Higher expressed genes in red, lower expressed genes in blue (n=2).

Figure S5

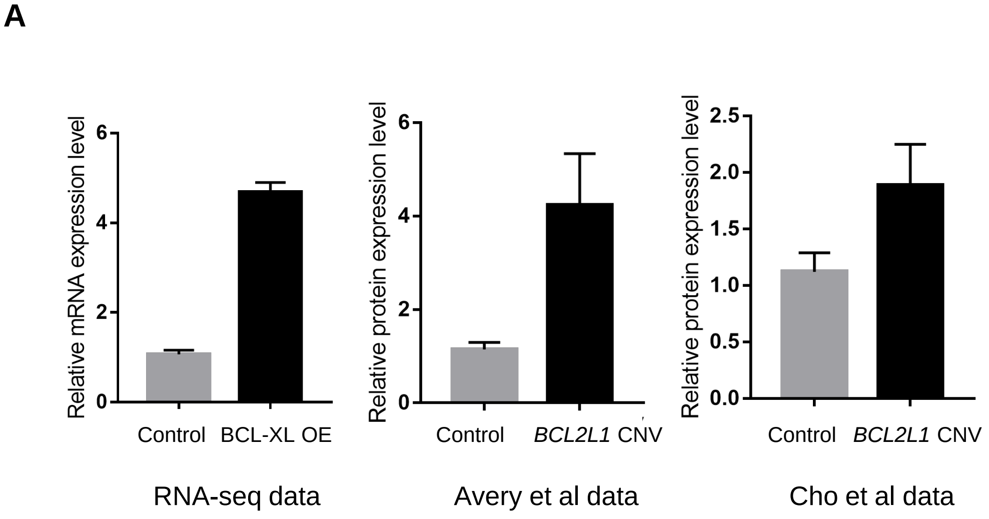

Figure S5. BCL-XL upregulation in BCL-XL OE or BCL2L1 CNV cells, Related to Figure 5. (A) Bar graph showing the level of BCL-XL transcript in OE H9 cells increased about 5 folds based on RNA-seq results. (B and C) Bar graph quantification of BCL-XL protein level based on western blot results from Avery et al 2013 and Cho et al 2018 (Avery et al., 2013; Cho et al., 2018) ($n \geq 2$).

Figure S6

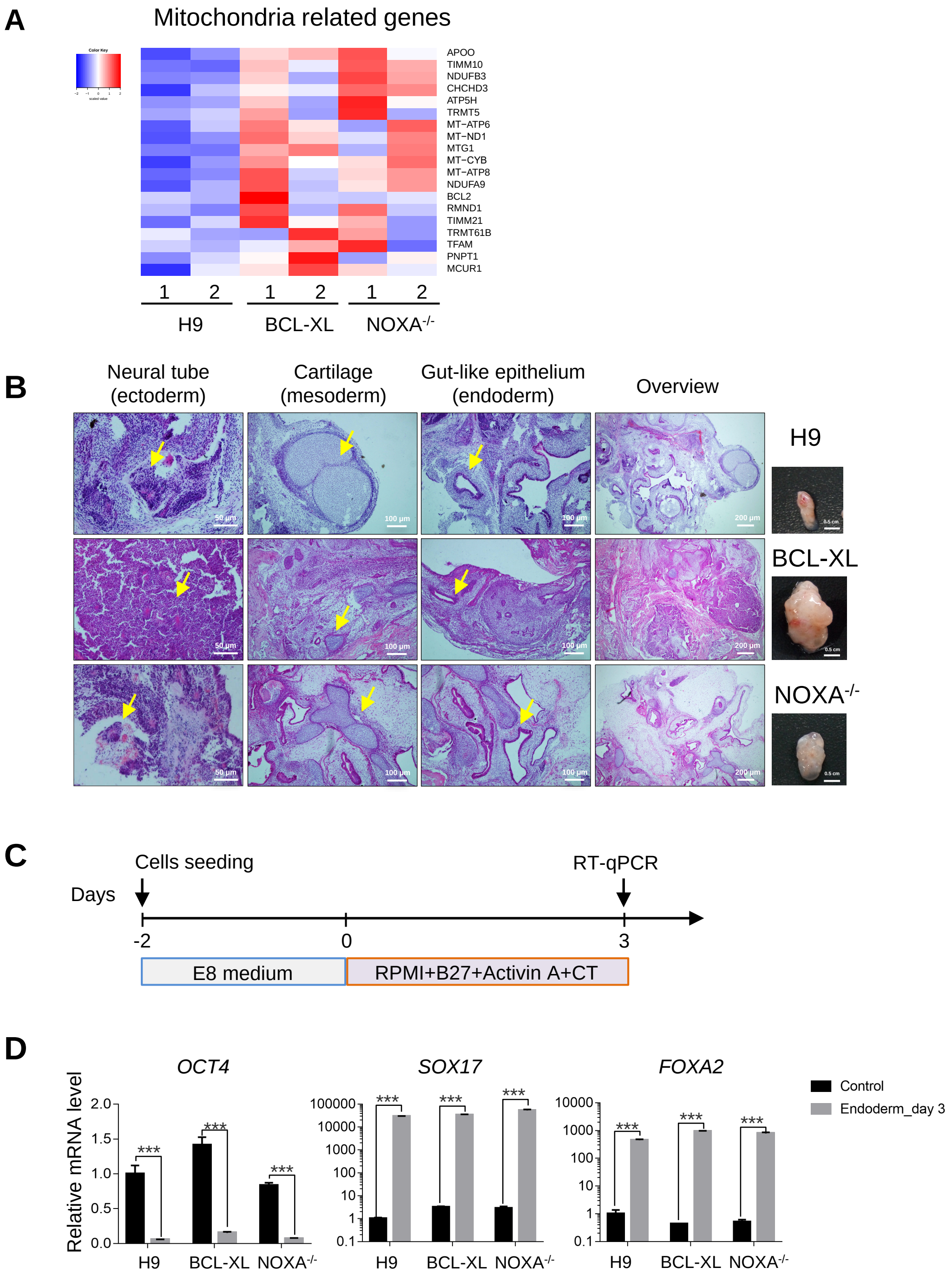

Figure S6. BCL-XL overexpression or NOXA knock out affect teratoma formation of hPSCs, Related to Figure 6.

(A) Heatmap showing commonly upregulated mitochondria genes in BCL-XL OE cells and NOXA$^{-/-}$ cells. FPKM values were scaled and color key indicated gene expression level. Higher expressed genes in red, lower expressed genes in blue (n = 2).

(B) Hematoxylin and eosin staining of teratoma sections from WT H9 cells, BCL-XL OE and NOXA knock-out cells. Three germ layers are indicated by yellow arrows.

(C) Schematic view of endoderm differentiation from hPSCs.

(D) RT-qPCR analysis of pluripotent genes (*OCT4*) and endoderm genes (SOX17 and FOXA2) at day 3 of differentiation (Data are represented as mean ± s.e.m. of 3 independent experiments, *** $P < 0.001$, based on unpaired Student's t-test).

Supplemental Experimental Procedures

KaryotypeAnalysis

Control and drug treated cells were cultured until confluent and sent for karyotyping at the Reproductive Medicine Center of the Peking University 3rd Hospital and Peking Union Medical College. Growing cells were treated with colcemid at 0.25 μg/ml for 4 h, then trypsinised to single cell suspension. Cells were resuspended in 0.4%M KCl hypotonic solution for 6 min at 37° C, followed by fixation with freshly made ice-cold methanol/acetic acid (3:1) for 20 min twice. After centrifugation, cells were resuspended in 300 μl fixation solution and dropped onto pre-cleaned glass slide. Slides were dried at 37° C overnight, followed by trypsin digestion and Giemsa staining. Images were taken on Zeiss Image M2 and analysed by SmartType software. For each group of cells, 50-70 chromosome spreads were imaged and analysed.

Flow Cytometry Analysis

To determine the cell cycle profile, hPSCs were incubated with 4 μg/ml Hoechst 33342 (Dojindo) for 2h, then washed and dissociated with 0.05% trypsin-EDTA. For cell death and apoptosis analyses, hPSCs were dissociated with 0.05% trypsin-EDTA, washed with PBS and then incubated with Alexa Fluor® 488 Annexin V/Dead Cell Apoptosis Kit (Invitrogen V13241 and V13245) for 15 min. For mitochondrial membrane potential detection, cells were stained with TMRE (Invitrogen) for 10 min or with JC-1 (Sigma). Flow cytometric analysis was carried out using a BD LSRFortessaTM FACS machine (BD Biosciences).

Immunostaining and Western Blot

For immunostaining, hPSCs were fixed in 4% PFA for 20 min, then permeabilized with 0.25% Triton X-100 for 20 min. Cells were then blocked with 5% FCS and incubated with primary antibody for 2 h, followed by washing in PBST and incubating with secondary antibody for 1h. For western blotting, hPSCs were lysed in SDS loading buffer. Protein samples were separated by gel electrophoresis and transferred to the nitrocellulose membrane, then incubated with primary antibody for 2 h and secondary antibody for 1 h. Antibodies used in this study were: TP53, NOXA and OCT3/4 (sc-126, sc-30209 and sc-5279 Santa Cruz), BCL-XL (ab32370 Abcam); MCL1, phospho-H3S10 and MCL1 (5453, 9706 and 9661 Cell Signaling Technology); HA (rat mAb 3F10, 1867423a Roche); GAPDH (CW0101A CWBiotech). Secondary antibodies used were: HRP-conjugated Goat Anti-Rat (ZSGB-Bio), HRP-conjugated Goat Anti-Rabbit (Cell Signaling Technology), HRP-conjugated Goat Anti-Mouse (Jackson ImmunoResearch), Dylight 488-conjugated Goat Anti-Mouse, Dylight 488-Goat Anti-Rabbit, Dylight 549-conjugated Goat Anti-Mouse and Dylight 549-conjugated Goat Anti-Rabbit (Thermo).

DNA Constructs and The Generation of Transgenic hPSC Lines

pCAG-H2B-mCherry-IRES-puro plasmid was constructed by ligating a fragment encoding H2B-mCherry in between a pCAG promoter and an IRES-puro resistance gene cassette in the pCAG-IRES-puro plasmid(Wang et al., 2008). TP53 knocking-down H9 cells were generated using the lentivirus pLKO.1 system. The targeted TP53 sequence is: 5'-GACTCCAGTGGTAATCTAC-3'. Empty vector was used as negative control. Lentivirus was packaged by transfecting 293FT cells with pLKO.1-shTP53 or pLKO.1 together with helper plasmids psPAX2 and pMD2.G-VSVG. Lentivirus was collected from supernatant by concentration. Stable shRNA transfectants were selected in a medium containing puromycin. For inducible BCL-XL overexpression H9 cells, human BCL-XL cDNA was cloned into a Tet-inducible piggyBac plasmid (Gift from Sanger Institute)(Wang et al., 2008) after a TRE3G promoter (Clontech). A 3xHA tag was added to the N-terminus of BCL-XL. A PGK promoter driving neomycin resistance gene cassette was also included in the same plasmid. TetON-3G transactivator (Clontech) was cloned into a separate piggyBac plasmid between a CAG promoter and an IRES-puro resistance gene cassette. H9 hESCs were transfected with PB-TRE3G-3HA-BCL-XL and PB-CAG-TetON-3G together with a plasmid expressing the piggyBac transposase (a gift from Sanger Institute) to facilitate stable integration. Stable clones were obtained by neomycin and puromycin double selection. Constitutive BCL-XL overexpressed H9 cell line was constructed with the pCAG-BCL-XL-IRES-Puro plasmid. H9 cells were transfected with the plasmid by electroporation and stable clones were obtained by puromycin selection.

NOXA Knock Out Using CRISPR

Cas9 nickase system was used to generate NOXA knock-out H9 cells in order to minimize the off-target effect. crRNAs targeting NOXA gene exon 1 and 2 were designed using the online Crispr design tool (http://crispr.mit.edu/). Two pair sgRNAs targeting NOXA exon1 and 2 were selected and ligated into pX335-U6-Chimeric_BB-CBh-hSpCas9n (D10A) Plasmid (Cong et al., 2013). H9 cells were transfected by electroporation. Single colonies were picked and expanded followed by genome PCR verification. The absence of NOXA protein was verified by western blot. The targeted sequences are 5'-ACGCTCAACCGAGCCCCGCGCGG-3' and 5'-TTCTTCCCAGGCATCTCCGCCGG-3', 5'-AGTAGGCCAGCGGTAATCTTCGG-3' and 5'-GTCATTAGACTG GGCGGCTGGG-3' in the downstream. To verify the deletion in NOXA gene, two primers were designed flanking the nicking site in exon2, so that clones with NOXA deletion would not have PCR products while clones with intact NOXA would have a 434bp DNA band. The primer sequences are: 5'- TGTGAAGGTGCATTCATGGG-3' and 5'-CAACAACAATGCACTGAACT-3'.

RNA Collection, High-throughput Sequencing and Data analysis

Cells were collected and washed by PBS. Then RNA was extracted with Trizol. 200ng RNA was used for reverse-transcription followed the SMART-seq2 protocol (Picelli et al., 2014). Then cDNA was fragmented by sonication using the Covaris sonicator. Fragmented cDNA was end-repaired, ligated with A base. After A base addition, the pre-indexed Illumina adapters were ligated. cDNA with adapters were enriched for another 11 cycles. Finally, the acquired cDNA libraries were sequenced on HiSeq X Ten platform.

Raw reads were trimmed with trim_galore. Then clean data were mapped to human reference genome(hg38) by STAR. Sequencing read coverage per gene was counted using HTSeq-count. FPKM values were calculated using Cufflinks. Differential gene expression analysis was performed using R packages "DESeq2" (Fold change >1.5, P value < 0.05). Subsequent gene ontology analysis was done by DAVID (https://david.ncifcrf.gov/summary.jsp). Gene set enrichment analysis was done with software downloaded online (http://software.broadinstitute.org/gsea/index.jsp) (Subramanian et al., 2007). PCA plot, heatmap and venn diagram were performed using R(http://www.r-project.org/).

Teratoma Analysis

All animal experiments were conducted in accordance with the Guide for the Care and Use of Animals for Research Purposes. The protocol of teratoma formation assay in severe combined immunodeficiency (SCID) mice was approved by Institutional Animal Care and Use Committee and Internal Review Board of Tsinghua University. One million wild type, BCL-XL OE or NOXA$^{-/-}$ H9 cells were injected into the groin region of the mouse (6 weeks old male and female mice were used without randomization). Two months later, teratomas were dissected, photographed, fixed with 4% PFA for 1 day, embedded in paraffin and sectioned at 10μm thickness to check differentiated tissue types. Experiments were repeated at least 3 times with more than 10 mice injected for each group.

Supplemental References

Cho, S.-J., Kim, K.-T., Jeong, H.-C., Park, J.-C., Kwon, O.-S., Song, Y.-H., Shin, J.-G., Kang, S., Kim, W., Shin, H.D., et al. (2018). Selective Elimination of Culture-Adapted Human Embryonic Stem Cells with BH3 Mimetics. Stem Cell Reports 11, 1–13.

Avery, S., Hirst, A.J., Baker, D., Lim, C.Y., Alagaratnam, S., Skotheim, R.I., Lothe, R. a, Pera, M.F., Colman, A., Robson, P., et al. (2013). BCL-XL mediates the strong selective advantage of a 20q11.21 amplification commonly found in human embryonic stem cell cultures. Stem Cell Reports 1, 379–386.

Wang, W., Lin, C., Lu, D., Ning, Z., Cox, T., Melvin, D., Wang, X., Bradley, A., and Liu, P. (2008). Chromosomal transposition of PiggyBac in mouse embryonic stem cells. Proc. Natl. Acad. Sci. 105, 9290–9295.

Cong, L., Ran, F.A., Cox, D., Lin, S., Barretto, R., Hsu, P.D., Wu, X., Jiang, W., and Marraffini, L.A. (2013). Multiplex Genome Engineering Using CRISPR/Cas Systems. Science (80-.). 339, 819–823.

Picelli, S., Faridani, O.R., Björklund, A.K., Winberg, G., Sagasser, S., and Sandberg, R. (2014). Full-length RNA-seq from single cells using Smart-seq2. Nat. Protoc. 9, 171–181.

Subramanian, A., Tamayo, P., Mootha, V.K., Mukherjee, S., Ebert, B.L., Gillette, M.A., Paulovich, A., Pomeroy, S.L., Golub, T.R., Lander, E.S., et al. (2007). Gene set enrichment analysis : A knowledge-based approach for interpreting genome-wide. Proc. Natl. Acad. Sci 102, 15545–15550.
